# Effectiveness and Safety of Peritoneal Dialysis Treatment in Patients with Refractory Congestive Heart Failure due to Chronic Cardiorenal Syndrome

**DOI:** 10.1155/2018/6529283

**Published:** 2018-05-17

**Authors:** Qiuyuan Shao, Yangyang Xia, Min Zhao, Jing Liu, Qingyan Zhang, Bo Jin, Jun Xie, Biao Xu, Rujun Gong, Chunming Jiang

**Affiliations:** ^1^Department of Nephrology, Affiliated Nanjing Drum Tower Hospital, Medical School of Nanjing University, Nanjing, China; ^2^Department of Cardiology, Affiliated Nanjing Drum Tower Hospital, Medical School of Nanjing University, Nanjing, China; ^3^Department of Medicine, University of Toledo College of Medicine, Toledo, OH, USA

## Abstract

**Aims:**

To evaluate the effectiveness and safety of peritoneal dialysis (PD) in treating refractory congestive heart failure (RCHF) with cardiorenal syndrome (CRS).

**Methods:**

A total of 36 patients with RCHF were divided into type 2 CRS group (group A) and non-type 2 CRS group (group B) according to the patients' clinical presentations and the ratio of serum urea to creatinine and urinary analyses in this prospective study. All patients were followed up till death or discontinuation of PD. Data were collected for analysis, including patient survival time on PD, technique failure, changes of heart function, and complications associated with PD treatment and hospitalization.

**Results:**

There were 27 deaths and 9 patients quitting PD program after a follow-up for 73 months with an average PD time of 22.8 ± 18.2 months. A significant longer PD time was found in group B as compared with that in group A (29.0 ± 19.4 versus 13.1 ± 10.6 months, *p* = 0.003). Kaplan–Meier curves showed a higher survival probability in group B than that in group A (*p* < 0.001). Multivariate regression demonstrated that type 2 CRS was an independent risk factor for short survival time on PD. The benefit of PD on the improvement of survival and LVEF was limited to group B patients, but absent from group A patients. The impairment of exercise tolerance indicated by NYHA classification was markedly improved by PD for both groups. The technique survival was high, and the hospital readmission was evidently decreased for both group A and group B patients.

**Conclusions:**

Our data suggest that PD is a safe and feasible palliative treatment for RCHF with type 2 CRS, though the long-term survival could not be expected for patients with the type 2 CRS. Registration ID Number is ChiCTR1800015910.

## 1. Introduction

Refractory congestive heart failure (RCHF) is a severe disease in clinical practice characterized by high mortality and low quality of life. Epidemiologic evidence indicates a growing prevalence of RCHF, probably associated with the improvement in managing various acute cardiovascular complications [1, 2]. It is now a major cause of death, hospitalization, and readmission in developed countries [3, 4]. Despite great advances in retarding the development of chronic congestive heart failure in the past decades, RCHF remains a therapeutic challenge with no effective treatment available yet except heart transplant.

Chronic renal dysfunction is a common finding in RCHF patients, either as a primary cause as seen in type 4 CRS or as a secondary consequence as seen in type 2 CRS. The presence of renal dysfunction is considered as one major risk factor for RCHF, since renal dysfunction is coupled with diminished kidney response to diuretics and accumulation of myocardial toxins which eventually precipitates clinical signs of heart failure [5]. Although mechanical ultrafiltration like hemofiltration has been used for acute decompensated heart failure with a favorable outcome, it is impossible to apply this therapy in the long term to treat RCHF with CRS due to hemodynamic complications, high cost, inconveniences, and vascular access-related problems [6]. Peritoneal dialysis (PD) [7, 8], a home-based therapy for uremia characterized by slow and gradual fluid removal, was first tried by Mailloux et al. to successfully treat nonuremic RCHF in 1964 [9]. Since then, various studies have been carried out to evaluate the safety and effectiveness of PD in treating RCHF. In contrast to a significant improvement in heart function and survival time for RCHF after PD treatment as shown by some studies, a lot of studies have failed to prove the benefit of PD for treating RCHF. For example, no significant survival benefit was found by Cnossen et al. [10] or Kunin et al. [11]. And substantial improvement in heart function was only seen in one of the five RCHF patients after PD in the study by Mailloux et al. [9]. Moreover, complications caused by PD itself such as leakage and dialysis-related infections may outweigh its potential benefit of improving heart function [12]. Thus, the clinical benefit of PD in RCHF treatment remains to be delineated.

It is now recognized that not all RCHF patients would benefit from PD treatment [13]. The exact reasons behind the disparity of response to PD treatment in RCHF are far from clear. However, the heterogeneity of RCHF may partly explain this disparity. While the diagnosis of RCHF is mainly based on the patient's clinical manifestation, the outcomes of PD treatment may vary among RCHF patients with different pathogenic mechanisms. Theoretically, several types of CRS such as type 2, 4, or 5 all can eventually contribute to RCHF. However, it is often difficult for clinicians to discriminate them clearly because of the complicated mutual effects between the heart and the kidney. Most previous studies [14, 15] have diagnosed type 2 CRS only based on patients' clinical presentations and thus may mistakenly include other types of CRS, resulting in inconsistent data. In view of these pitfalls, this prospective study was designed to determine the role of PD in treating RCHF with type 2 CRS, which was diagnosed according to both clinical presentations and objective laboratory tests, such as the ratio of serum urea to creatinine and urinary analyses, to exclude other types of CRS.

## 2. Patients and Methods

### 2.1. Patients and Groups

This prospective study was approved by the Ethics Review Board of the Drum Tower Hospital affiliated with Nanjing University Medical School. Patients with RCHF and no contraindications for PD therapy were enrolled from 1 January 2007 to 31 December 2010 and received PD treatment. All patients signed the informed consent. The initiation of a PD program was decided by both nephrologists and cardiologists when the symptoms of heart failure cannot be controlled effectively by all other available treatments.

The inclusion criteria are as follows: (1) their age is above 18 years; (2) the extent of heart failure is assessed as stage III or IV based on the New York Heart Association (NYHA) functional classification; (3) heart function cannot be improved or sustained despite maximal conservative treatment including large dose of diuretics; (4) recurrent hospitalization is due to heart failure caused by volume overload (at least twice in the preceding 12 months). The exclusion criteria are as follows: (1) patients suffered from an acute deterioration of heart and/or renal function; (2) estimated glomerular filtration rate (eGFR) calculated by Modification of Diet in Renal Disease (MDRD) equation is less than 10 mL/min/1.73 m^2^ [16]; (3) ratio of serum urea to creatinine concentrations is less than or equal to 10. Totally, 36 patients met our criteria and were included in this study during the enrollment period. The follow-up period began from the PD initiation until the cessation of PD or death, whichever occurred first. The study was ended in September 2016 when the last patient quit the PD treatment.

Patients were divided into two subgroups for study: group A (*n* = 14) was defined as type 2 CRS whose ratio of serum urea to creatinine concentrations was more than 20 and in absence of obvious hematuria and/or proteinuria; the other 22 patients (group B) were defined as non-type 2 CRS.

### 2.2. Data Collection

Patients' demographics, primary disease, comorbidity, and other baseline data were recorded at the initiation of PD. After PD treatment, body weight, residual renal function, urinary volume, blood pressure, biochemical test, ultrafiltration volume, and heart function were evaluated every 3 to 6 months. Dialysis regime, complications, survival time, reasons for technical failure and death, and readmissions with its causes were documented timely for analysis. Patients' residual renal function (RRF) was calculated based on urea and creatinine levels in serum and 24-hour urine collections.

### 2.3. Treatment Strategies

PD therapy was initiated immediately after PD catheter insertion without break-in period and using 2 L exchange volume with varying dwell times and cycles, depending on the patients' fluid status. Fluid balance indicated by patient's dry body weight was one of the key goals of long-term PD treatment. The dry body weight was reevaluated by physicians every 6 months. Patients were asked to monitor their daily body weight using a home-used electronic scale. If a patient's body weight had increased beyond 3% of dry body weight, a stepwise approach aiming at fluid removal was introduced as follows in sequence: it increased the oral dosage of furosemide up to 240 mg per day unless anuria was present and then increased the frequency of PD exchanges with short dwell time with the minimal dwell time of 1 hour per cycle, and at last higher osmatic peritoneal dialysis fluid was applied with appropriate dwell time. Those whose dry body weight cannot be achieved by above interventions would be readmitted for further treatment.

In addition to adequate fluid removal, dialysis dosage during follow-up was also adjusted to attain a total weekly KT/V of no less than 1.7. ARB or ACEI and *β*-receptor blocker were regularly prescribed to our patients after PD initiation if no contraindication existed. The prescriptions of other drugs, such as antihypertension, erythropoietin, vitamin D3, and iron agents, were individualized according to KDIGO guidelines.

## 3. Statistical Analysis

Continuous variables were presented as the mean ± standard deviation (SD) or median (interquartile range). Qualitative data were expressed as absolute numbers and percentages. Wilcoxon signed-rank test, Mann–Whitney test, paired or independent sample *T*-tests, or *χ*^2^ test were used as appropriate. Patient survival time on PD and technique survival probability were of particular interest in present study and were evaluated by the Kaplan–Meier test. Patient survival time on PD was defined from the beginning of PD to all-cause death for the patients, who died during PD or less than 3 months after conversion to other treatments. Patients, who survived more than 3 months after the cessation of PD treatment, were considered as censor data. Technique failure was defined as the cessation of PD therapy for any catheter-related complications and censored by renal transplantation and conversion to hemodialysis or death. Stepwise multivariate regression was applied to identify the independent predictors for patient survival time on PD treatment. All statistical analyses were performed with SPSS software application (version 20.0 for Windows: SPSS Institute, Chicago, IL, USA). All tests were two-tailed, and a value of *p* < 0.05 was considered statistically significant.

## 4. Results

### 4.1. Patient Characteristics

Table 1 shows the characteristics of all studied patients. There were significant differences between type 2 CRS (group A) and non- type 2 CRS (group B) groups in patient age (*p* = 0.023), ischemic heart disease (*p* = 0.036), baseline eGFR (*p* < 0.001), systolic and diastolic blood pressure (both *p* < 0.001), the use of angiotensin converting enzyme inhibitor (ACEI), and angiotensin II receptor blockers (ARB) (*p* = 0.0491). There were no significant differences in dyslipidemia, body mass index (BMI), anemia, serum albumin, and arrhythmias. The use of beta-blockers, diuretics, and statins was not statistically different between two groups of patients (*p* > 0.05).

### 4.2. Patient Survival and Death

After a follow-up for 73 months, all patients reached the end point with 27 deaths and 9 patients being converted to hemodialysis due to various reasons. The leading causes of death for group A were acute decompensated heart failure and sudden death with an incidence of 35.7% and 28.6%, which was significantly higher than that in group B (9.09% and 4.55%, respectively, both *p* < 0.05). Other causes for death were myocardial infarction, stroke, infection, and unknown reasons, which were not significantly different between the two groups.

**Figure 1:**
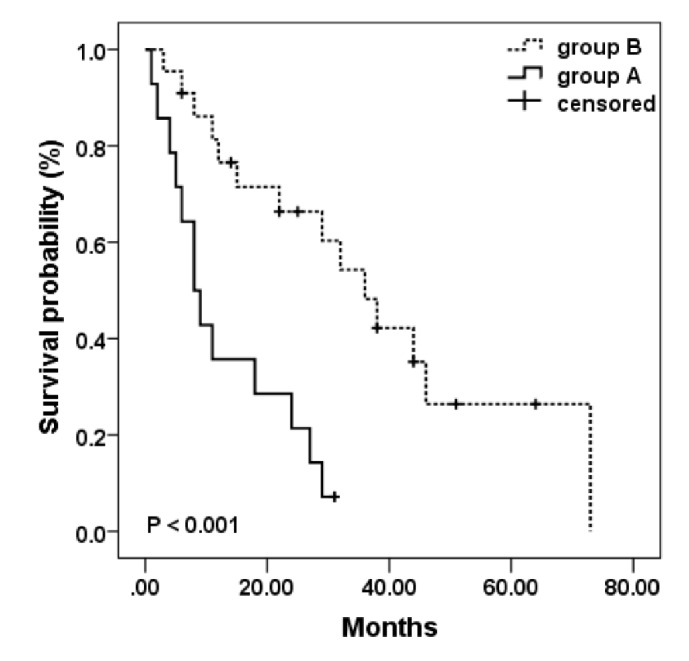
Kaplan–Meier estimates of cumulative survival probability in PD patients.

The average PD duration was 22.8 ± 18.2 months. A significant longer PD time was found in group B as compared with that in group A [29.0 ± 19.4 (medium: 27, range: 3–73) versus 13.1 ± 10.6 (medium: 8.5, range: 1–31) months, *p* = 0.003].  Figure 1 shows Kaplan–Meier curves for overall life survival on PD for group A and B patients (*p* < 0.001).

Multivariate predictors of patient survival time on PD are shown in Table 2. Variables included in the final analysis were CRS groups, ages, history of ischemic heart disease, systolic and diastolic blood pressure, baseline eGFR, LVEF, and ACEI/ARB use, which have a *p* value less than 0.1 in univariate analysis. It seems that type 2 CRS is the most important negative predictor for patient survival time on PD. Other independent predictors are patient ages and history of ischemic heart disease.

### 4.3. Changes of Clinical and Laboratory Parameters 6 Months after PD

There were 28 patients who lived more than 6 months after PD initiation.  Table 3 shows the changes of key clinical parameters before and 6 months after PD.

Both groups of patients showed significant decrease in body weight after PD, while more body weight loss was seen in group B than in group A (*p* = 0.002). A significantly deteriorated renal function and decreased systolic blood pressure were found in group B (both *p* < 0.05), but not in group A. The daily urine output significantly increased in group A (*p* = 0.022), whereas a marked decrease was found in group B (*p* = 0.027). At 6 months after PD, the daily peritoneal ultrafiltration was 198 ± 206 mL for group A, which is significantly lower than that for group B (860 ± 306 mL, *p* < 0.001).

Totally, 18 patients in group B experienced an improvement in heart function as assessed by NYHA classification. This is significantly greater than the number of patients in group A (*p* = 0.047). After 6-month PD therapy, the levels of B-type Natriuretic Peptide (BNP) in both group patients were decreased (both *p* < 0.001). The improvement of left ventricular ejection fractions (LVEF) was seen in group B patients (*p* < 0.001), but not in group A (*p* = 0.162).

### 4.4. PD Related Compilations

**Figure 2:**
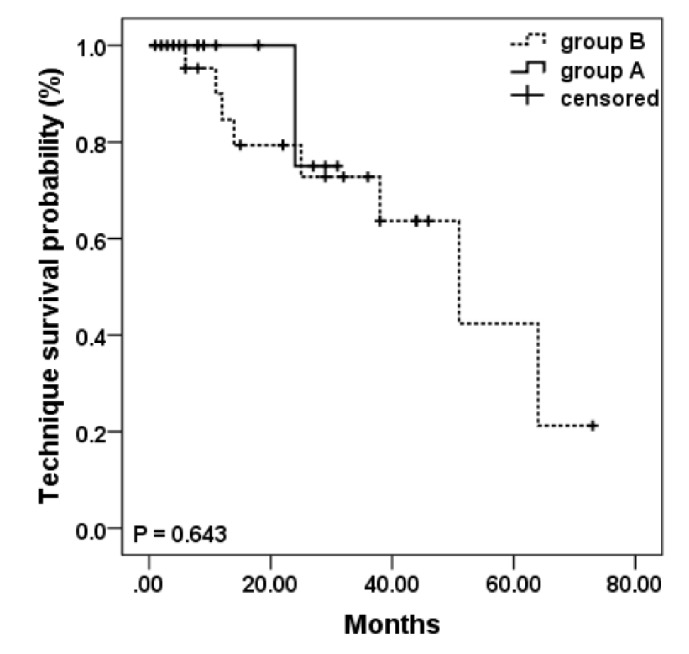
Kaplan–Meier estimates of cumulative technique survival probability in PD patients.

During the follow-up, one patient (2.78%) in group B experienced catheter malfunction and eventually was converted to HD treatment. There was 1 case of (2.78%) slight catheter leakage that occurred at the early stage of PD. However, the leakage was stopped by reducing dwell volume in supine posture after several days. Among the 15 episodes of peritonitis, 14 happened in group B corresponding to a rate of 0.26 times per patient-year, which is significantly higher than the rate of 0.09 times per patient-year in group A (*p* = 0.023). The causative organisms for the peritonitis in group A were tested to be fungi, while those for group B patients were* Staphylococcus epidermidis* (8 episodes), Gram-negative bacilli (3 episodes), fungi (1 episode), and negative culture (2 episodes). The overall technique survival probability of the two group patients was not significantly different as shown in Figure 2 (*p* = 0.643).

### 4.5. Hospital Readmission

There were 65 readmissions for all patients during follow-up for various reasons (Table 4). Among them, 56 times were for group B with a rate of 1.05 per patient-year and 9 times for group A with a rate of 0.812 per patient-year (*p* = 0.026). Higher incidence of PD-associated readmissions was seen in group B than that in group A (0.319 versus 0.090 per patient-year, *p* = 0.023). Peritonitis occurred more frequently in group B patients than that in group A (0.263 versus 0.090 per patient-year, *p* = 0.025). No significant difference was found in overall cardiovascular- or pneumonia-associated readmission between the two groups (*p* > 0.05).

## 5. Discussion

The effectiveness and safety of PD in RCHF treatment are still controversial. In this prospective study, RCHF patients with type 2 CRS received PD treatment and demonstrated that (1) the benefit of PD on the improvement of survival and LVEF was limited; (2) the impairment of exercise tolerance indicated by NYHA classification was markedly improved; (3) the technique survival was remarkably high with low rate of PD-associated complications; (4) the hospital readmission was evidently decreased. Our data suggest that PD is a safe and feasible alternative treatment for RCHF with type 2 CRS.

RCHF has a notorious prognosis as about three-fourth patients would die in 12 months. Recently, some researchers reported PD as a potential promising modality in treating RCHF with type 2 CRS in either case reports or observational studies [9, 12, 17]. However, it is still a hot topic of dispute as discrepant conclusions have been drawn from different trials [12, 18]. Although some studies demonstrated a striking improvement in 1-year survival rates to as high as 82% following PD therapy, some dismal results have also been reported. For example, Cnossen et al. [10] examined 24 type 2 CRS patients with non-end-stage renal failure treated by PD and found that only 3 (12.5%) patients have lived longer than 12 months. Kunin et al. [11] also found only 4 of 37 RCHF patients have survived more than 2 years after PD treatment. Consistent with these findings, the RCHF patients with type 2 CRS in this study also exhibited a poor survival rate with about one-third patients lived longer than 1 year and one-fifth more than 2 years. This was significantly lower than those with non-type 2 CRS, whose 1-year survival was more than 80% and 2-year survival more than 50%. We hypothesized that this limited survival benefit acquired from PD treatment for type 2 CRS patients could be probably due to the severely impaired myocardium.

In the present study, we found that type 2 CRS group is an independent predictor for patient survival time after PD, suggesting that not all CRS would equally benefit from PD treatment. In addition to CRS types, other independent risk factors for short PD survival time include old ages and history of ischemic heart disease and this is consistent with previous studies. However, other important risk factors associated with patient survival have failed to be identified here. For example, insulin-supplement therapy has recently been reported to significantly be associated with patient mortality in elderly patients with heart failure [19]. The explanation for this may be the limited statistical power of this study due to the small sample size. Future large scale and multicenter studies are merited for detecting more relevant factors associated with the risks for death.

The survival probability for RCHF patients with type 2 CRS in this study was apparently lower than that reported in most previous studies [13, 20–23]. The reason for this finding is complicated. First, the difference in heart disease severity as well as primary etiology of heart diseases may contribute to significant variance of life expectance. In fact, the mean LVEF levels reported in previous studies vary from 22% to as high as 42% [24–27]. The mean LVEF level in this study was 24.6% which is lower than in most previous studies. Secondly, the residual renal function is one of the key determinants of the outcome of patients with type 2 CRS. However, the levels of eGFR included in published studies were strikingly disparate, ranging from 10.5 to 49 mL/min/1.73 m^2^. Thirdly, most previous studies identified type 2 CRS only according to clinical symptoms without applying any objective parameter [14, 15], and this cannot rule out the possibility that patients with other types of CRS or mixed CRS were mistakenly categorized as type 2 CRS. Just as shown in this study, the patients with non-type 2 CRS responded to PD treatment much better than those type 2 CRS patients. Thus, the incorrect inclusion of other types of CRS as type 2 CRS would largely overestimate the effectiveness of PD treatment.

Consistent with the less benefit on patients' survival, the improvement of LVEF after PD in the present study was also unremarkable for type 2 CRS patients. However, striking elevation of LVEF was seen in the non-type 2 CRS patients after 6-month PD treatment. This difference in the change of LVEF after PD treatment indicates a more prominent irreversible damage in type 2 CRS patients than in non-type 2 CRS patients, as evidenced by more impaired tolerance to PD ultrafiltration and lower blood pressure levels in the type 2 CRS patients. In contrast to negligible improvement in LVEF after PD, improvement in NYHA class, as indicated by an increase in exercise tolerance, was experienced in most type 2 CRS patients. Moreover, almost all patients with type 2 CRS demonstrated a diminished serum BNP levels following PD treatment [12]. These findings in the present study infer that PD therapy can help type 2 CRS patients ameliorate fluid overload and thereby improve clinical symptoms.

One major concern about the application of PD in RCHF is the potential high rates of technique failure and PD-associated complications, such as peritonitis and catheter malfunction. However, thanks to the recent advances in PD, the technique survival of PD is now considerably satisfactory [28, 29]. Likewise, our study showed excellent technique survival in type 2 CRS patients: only 1 patient dropped out of PD therapy due to peritonitis. By the adoption of the Y connection systems equipped with the “flush before fill” design in combination with the routine prevention and treatment for peritonitis, both the rate of peritonitis and the associated death were markedly reduced. As shown in this study, during follow-up of the entire study period, a rate of peritonitis was only 0.09 per patient-year. Notably, the rate of peritonitis was significantly lower in type 2 CRS patients than that in non-type 2 CRS patients. This might be secondary to the low daily dosage prescribed for type 2 CRS patients, as shorter period of PD fluid exchange would surely decrease the risk of contamination during operation. Moreover, more intensive prophylaxis treatment given to type 2 CRS patients for the severity of disease might also be the important factors for their low rate of peritonitis. Leak was an early complication after PD catheter surgery especially when short break-in time before dialysis has been applied. However, only 1 slight leak was found for our CRS patients with no significant adverse response to the treatment procedure. One possible reason for the low rate of leak in CRS patients may be associated with the preexisting large volume ascites before PD. After the PD initiation, the abdominal pressure was not increased but markedly decreased. Nevertheless, high rate of leak has been reported in previous studies [30, 31]. Thus, a high-quality surgery was definitely most crucial in preventing early leak.

In line with most published studies, heart-associated hospital readmission was significantly decreased in this cohort of CRS patients [31–33]. However, the readmission for heart complications was more frequent, though not statistically significant, for type 2 CRS than for non-type 2 CRS patients. This is probably due to the difference in the severity of heart disease between the two group patients. The total readmission in this study was decreased evidently with a rate of 0.812 per patient-year for type 2 CRS patients. Our data indicated that the beneficial effect of PD treatment on decreasing total readmission could not be countered-back by the additional readmission for PD-associated complications, which is contrary to some previous reports. Interestingly, we found that the total readmission was decreased more significantly for type 2 CRS patients than that for non-type 2 CRS patients. In addition to the above-mentioned risk factors for high incidence of peritonitis in non-type 2 CRS patients, like long-term treatment and frequent daily exchanges, fluid overload without dyspnea was another potential reason for the high readmission of the non-type 2 CRS patients. The remarkable decline of residual renal function in non-type 2 CRS patients after PD due to either the progression of renal disease or the aggressive ultrafiltration may make their volume control difficult during the long-term follow-up. Consistently, daily urine output in non-type 2 CRS patients was significantly reduced after 6-month PD treatment as compared with that prior to PD initiation. In contrast, an evident increase of urinary volume was found in type 2 CRS patients following PD therapy.

Our study has some strength as compared with previous studies. First, patients with type 2 CRS in this study were recruited based on the combination of both clinical symptoms and laboratory parameters. This can largely avoid the misclassification of other types of CRS or mixed CRS that may largely overestimate the efficacy of PD treatment. Second, all patients enrolled in this study were observed till death or till quitting PD program. This long-term follow-up would allow the collection of more comprehensive information on the effectiveness and safety of PD in type 2 CRS than previous short-term studies. However, we also acknowledge that the present study is not without limitations. First, as a nonrandomized control study, the results from this study should be further verified by further investigations. Second, the cut-off value of 20 for the ratio of serum urea to creatinine concentrations that was adopted in this study to discriminate type 2 CRS from other types of CRS is totally empirical with regard to the differentiation of prerenal and intrinsic acute kidney injury. The specificity and sensitivity of this index in the diagnosis of type 2 CRS remained to be defined. This issue could be resolved if some specific biomarkers for type 2 CRS are developed in the future. Last but not least, the lack of a group treated by mechanical ultrafiltration makes it impossible here to prove PD as the most optimal method to treat RCHF with type 2 CRS. In-depth investigation is required.

In conclusion, we demonstrated that PD therapy is only palliative, with no long-term survival benefit for RCHF patients with type 2 CRS. Our data suggested that PD is a safe and feasible therapeutic modality for patients with type 2 CRS when improvement in clinical symptoms, rather than long-term survival, is the main pursuing goal of treatment. Large scale, multicenter, randomized control studies are certainly merited to validate our findings in this study.

## Figures and Tables

**Table 1:** Characteristics of study patients.

Characteristic	Group A (*n* = 14)	Group B (*n* = 22)
*Age (years)*	53.6 ± 15.4	68.3 ± 12.7^*∗∗*^
*Men (%)*	8 (57.1)	14 (63.6)
*Diabetes mellitus (%)*	5 (35.7)	12 (54.5)
*Body mass index*	21.0 ± 1.98	22.4 ± 1.89
*Blood urea/creatinine*	36.8 ± 10.2	14.7 ± 3.03^*∗∗*^
*Cardiomyopathy*		
Ischemic	6 (42.9)	17 (77.3)^*∗*^
Rheumatic	3 (21.4)	1 (4.54)
Idiopathic	5 (35.7)	2 (9.09)^*∗*^
Others/unknown	-	2 (9.09)^*∗*^
*Potential causes of renal damage other than heart failure*		
Diabetic kidney disease	1 (7.14)	10 (45.5)^*∗*^
Chronic glomerular nephritis	-	3 (13.6)
Hypertensive glomerular sclerosis	8 (57.1)	3 (13.6)
Others/unknown	-	6 (27.3)
*NYHA stage*		
III	6 (42.9)	9 (40.9)
IV	8 (57.1)	13 (59.1)
*eGFR (mL/min/1.73 m* ^*2*^)	27.8 ± 9.87	15.1 ± 3.51^*∗∗*^
*Hemoglobin (g/dL)*	92.0 ± 14.5	86.5 ± 11.4
*Serum cholesterol (mmol/L)*	4.08 ± 1.01	4.84 ± 1.89
*Serum triglyceride (mmol/L)*	2.55 ± 1.34	2.98 ± 1.01
*Serum albumin (g/L)*	33.2 ± 4.12	32.4 ± 4.81
*Systolic blood pressure (mmHg)*	115 ± 16	159 ± 24^*∗∗*^
*Diastolic blood pressure (mmHg)*	64 ± 18	89 ± 19^*∗∗*^
*Atrial fibrillation*	3 (21.4)	8 (36.4)
*Prolonged Q-Tc interval*	6 (42.9)	9 (40.9)
*Drug prescription after PD (%)*		
ACEI/ARB	9 (64.3)	20 (90.1)^*∗*^
Beta-blockers	12 (85.7)	21 (95.5)
Diuretics	11 (78.6)	22 (100)
Statins	*13 (92.9)*	*20 (90.1)*
Aspirin	*8 (57.1)*	*13 (59.1)*
Erythropoietin	*2 (14.3)*	*8 (36.4)*
Insulin (% of DM)	*4 (80)*	*12 (100)*

*Note*. Compared with group A ^*∗*^*p* < 0.05; ^*∗∗*^*p* < 0.01. eGFR: estimated glomerular filtration rate.

**Table 2:** Multivariate predictors for patient survival time on PD.

	B	SE	*β*	95% CI	*p* value
Group B versus group A	28.927	3.990	0.814	20.801, 37.053	0.000
Ages	−0.583	0.157	−0.512	−0.903, −0.264	0.001
Ischemic versus nonischemic heart disease	−12.717	4.668	−0.353	−22.225, −3.208	0.010

**Table 3:** Clinical and laboratory characteristics of study patients prior to and 6 months after PD.

	*Group A* (*n* = 9)		*Group B* (*n* = 19)	
	Before	After	Before	After
Weight (kg)	62.4 ± 8.84	59.9 ± 7.82^*∗∗*^	64.6 ± 10.5	57.7 ± 9.57^*∗∗*^
ΔWeight (kg)	-	2.59 ± 1.15	-	6.93 ± 3.81^##^
SBP (mmHg)	114 ± 14.7	109 ± 9.38	159 ± 26.4	143 ± 20.7^*∗*^
DBP (mmHg)	66.1 ± 18.0	63.3 ± 8.44	89.2 ± 18.8	80.5 ± 16.9
Residual renal function (mL/min/1.73 m^2^)	25.0 ± 5.27	26.9 ± 5.39	14.1 ± 2.85	9.98 ± 3.31^*∗*^
Hemoglobin (g/L)	89.7 ± 12.0	102.7 ± 20.8^*∗∗*^	85.1 ± 12.4	103 ± 16.5^*∗∗*^
Albumin (g/L)	31.2 ± 3.07	27.6 ± 4.06^*∗*^	32.4 ± 4.59	32.9 ± 3.38
Urine output (mL/day)	865 ± 225	1021 ± 178^*∗*^	1010 ± 416	781 ± 357^*∗*^
Peritoneal ultrafiltration (mL/day)	-	198 ± 206	-	860 ± 306^##^
Dialysis dosage (L/day)	-	2 (2, 4)	-	6 (4, 8)^##^
NYHA class				
* II*	0	5^*∗*^	0	13^*∗∗*^
* III*	5	2	8	5
* IV*	4	2	11	1
BNP (ng/mL)	2483 ± 1364	807 ± 498^*∗∗*^	3013 ± 1566	412 ± 238^*∗∗*^
LVEF (%)	24.6 ± 3.78	27.0 ± 6.12	32.0 ± 9.92	48.5 ± 4.32^*∗∗*##^

*Note*. Compared with before ^*∗*^*p* < 0.05; ^*∗∗*^*p* < 0.01; compared with group A ^##^*p* < 0.01. Data are expressed as numbers (percentage). SBP: systolic blood pressure; DBP: diastolic blood pressure; LVEF: left ventricular ejection fractions; BNP: B-type natriuretic peptide.

**Table 4:** Reasons for readmission.

	Group A	Group B
*Total readmissions*	*9 (0.812)*	*56 (1.05)* ^*∗*^
*PD associated*	*1 (0.090)*	*17 (0.319)* ^*∗*^
Catheter malfunction	0	1 (0.019)
Peritonitis	1 (0.090)	14 (0.263)^*∗*^
Tunnel related	0	2 (0.038)
*Cardiovascular associated*	*5 (0.451)*	*12 (0.225)*
ACS	1 (0.090)	4 (0.075)
Heart failure	2 (0.180)	6 (0.113)
Stroke	1 (0.090)	2 (0.038)
*Pneumonia*	*2 (0.180)*	*6 (0.038)*
*Fluid overload without dyspnea*	*1 (0.090)*	*14 (0.263)*
*Others*	*0*	*7 (0.131)*

*Note*. Compared with group A ^*∗*^*p* < 0.05. Data are expressed as numbers (rate of incidence: times per patient-year). Mann–Whitney test was applied. PD: peritoneal dialysis; ACS: acute coronary syndrome.
